# Reduced Expression of Brain-Enriched microRNAs in Glioblastomas Permits Targeted Regulation of a Cell Death Gene

**DOI:** 10.1371/journal.pone.0024248

**Published:** 2011-09-02

**Authors:** Rebecca L. Skalsky, Bryan R. Cullen

**Affiliations:** Department of Molecular Genetics and Microbiology and Center for Virology, Duke University Medical Center, Durham, North Carolina, United States of America; University of Valencia, Spain

## Abstract

Glioblastoma is a highly aggressive malignant tumor involving glial cells in the human brain. We used high-throughput sequencing to comprehensively profile the small RNAs expressed in glioblastoma and non-tumor brain tissues. MicroRNAs (miRNAs) made up the large majority of small RNAs, and we identified over 400 different cellular pre-miRNAs. No known viral miRNAs were detected in any of the samples analyzed. Cluster analysis revealed several miRNAs that were significantly down-regulated in glioblastomas, including miR-128, miR-124, miR-7, miR-139, miR-95, and miR-873. Post-transcriptional editing was observed for several miRNAs, including the miR-376 family, miR-411, miR-381, and miR-379. Using the deep sequencing information, we designed a lentiviral vector expressing a cell suicide gene, the herpes simplex virus thymidine kinase (HSV-TK) gene, under the regulation of a miRNA, miR-128, that was found to be enriched in non-tumor brain tissue yet down-regulated in glioblastomas, Glioblastoma cells transduced with this vector were selectively killed when cultured in the presence of ganciclovir. Using an in vitro model to recapitulate expression of brain-enriched miRNAs, we demonstrated that neuronally differentiated SH-SY5Y cells transduced with the miRNA-regulated HSV-TK vector are protected from killing by expression of endogenous miR-128. Together, these results provide an in-depth analysis of miRNA dysregulation in glioblastoma and demonstrate the potential utility of these data in the design of miRNA-regulated therapies for the treatment of brain cancers.

## Introduction

Glioma is an aggressive, malignant brain tumor involving glial cells. Each year in the United States alone, over 20,000 new patients are diagnosed with glioma and 13,000 patients die [1]. The most severe and malignant form, glioblastoma multiforme, is highly infiltrative, rapidly growing, and accounts for 52% of all brain tumors. Despite advances in chemotherapy, radiation and surgery, patient prognosis remains poor, with a median survival time of 14 months [1]–[3]. Thus, identifying new therapeutic strategies for glioblastoma by understanding the molecular pathology of this disease has become a major research focus.

Glioblastoma is associated with a number of mutations at the genomic level including mutations in the PTEN tumor suppressor and amplification of the gene encoding the epidermal growth factor receptor (EGFR) [3], [4]. More recently, dysregulated microRNA (miRNA) expression, such as over-expression of the anti-apoptotic miRNA miR-21, has been linked to tumor pathogenesis [5].

MiRNAs are ∼22 nt non-coding RNAs that post-transcriptionally down-regulate cellular gene expression by binding to complementary sequences generally located in the 3′ untranslated regions (UTRs) of target mRNAs. Target specificity is predominantly determined by nucleotides 2-8 of the mature miRNA, the miRNA “seed,” which generally exhibits full sequence complementarity to target mRNAs. Over 1400 human miRNAs have been identified to date [6], several of which exhibit altered expression patterns in many human cancers, including glioblastoma, and are thought to positively or negatively regulate cancer progression [5], [7]–[11]. Post-transcriptional regulation of gene expression by miRNAs is complex, as a single miRNA species can regulate multiple mRNAs, while a single mRNA can be targeted by different miRNAs. In fact, individual miRNAs are predicted to target as many as 200 different mRNAs [12]. Moreover, single pre-miRNA precursors occasionally give rise to not only the dominant, miRNA strand, but also a less highly expressed passenger or star strand, which may also be able to regulate mRNA expression [13].

Previous miRNA profiling studies, based on miRNA microarrays and PCR arrays, have revealed a number of miRNAs to be altered in gliomas [9], [14]–[16]. In addition to miR-21 [5], the pro-oncogenic miRNA miR-10b is upregulated in glioblastomas and has recently been shown to be a significant contributor to tumor growth in vivo [9], [17]. MiRNAs down-regulated in gliomas include miR-7, miR-124, miR-128, miR-137, and miR-181a/b [9], [14], [15], [18], [19]. miR-128, miR-124, and miR-137 are all enriched in the brain and have been shown to regulate neuronal differentiation, maturation, and/or survival [15], [20]–[23]. Of note, miR-7 can directly repress expression of EGFR, which is often amplified at the genetic level and/or over-expressed at the protein level in gliomas [19].

In this study, we profiled global miRNA expression levels in six adult glioblastomas and three non-tumor brain tissue samples using high-throughput sequencing. In contrast to microarrays and RT-PCR arrays, high-throughput sequencing permits the detection of not only the dominant mature miRNA species (i.e. 5p versus 3p) but also potentially important sequence variations that may occur, such as 5′ nucleotide additions, which affect seed-based targeting, and RNA editing, which alters the targeting capacity of the mature miRNA [24]–[27]. Our analysis identified at least 20 miRNAs and miRNA star strands (miRNA*) that are significantly differentially expressed. In addition, we identified variation in the 5′ ends of several miRNAs expressed in glioblastomas as well as editing in the seed region, which should modulate the mRNA targets bound by these miRNAs. This information can now be combined with mRNA expression profiles to identify potential brain-specific target mRNAs.

Our second objective was to use our miRNA deep sequencing data to facilitate the design of a miRNA-regulated gene therapy vector. Our aim was to permit the expression of a cell suicide gene, namely herpes simplex virus thymidine kinase (HSV-TK), in glioma cells while limiting off-target effects by ablating its expression in neurons and other important cell types in the brain. Therefore, we focused on miRNAs that are down-regulated in glioblastoma compared to non-tumor brain tissue. By exploiting naturally occurring brain-specific miRNAs, we hypothesized that the incorporation of specific miRNA target sites into the 3′ untranslated region (UTR) of the HSV-TK open reading frame would effectively restrict its expression in non-transformed cells. Similar approaches have been used to selectively prevent transgene expression in hematopoetic cells and other cell types [28]–[31]. We engineered artificial target sites for miR-128, which is highly down-regulated in glioblastoma, into the 3′UTR of HSV-TK. Both the ectopic expression of miR-128 in glioblastoma cells and the natural, endogenous level of miR-128 expression seen in neuronal cells indeed limited cell death when compared to control glioblastoma cells. These results demonstrate the significant potential of miRNA-regulated therapies in the treatment of glioblastoma.

## Results

### Small RNA deep sequencing reveals differences in human miRNA expression patterns

Using high-throughput sequencing, we investigated miRNA expression patterns in six human glioblastomas and three non-tumor brain tissue samples. Small RNA populations corresponding to 18–25 nt in length were sequenced using an Illumina GAII analyzer. Between 1.8 and 4.9 million reads were obtained per sample (Table S1) and the sequence length distributions showed peaks at ∼22–23 nt for each sample, corresponding to the average length of a mature miRNA (Fig. S1). To first determine the complete miRNA transcriptome of glioblastomas, we pooled reads from all six samples and aligned them to all human and viral miRNAs/miRNA*s present in Sanger miRBase v 16.0. Among the 16,305,416 mapped reads, 484 mature human miRNAs/miRNA*s were represented at least 10 times (Table S2).

The most abundant miRNAs in both glioblastomas and non-tumor brain tissues were members of the let-7 family, which accounted for ∼23% of the total reads (Table S2, S3). miR-21, miR-9, miR-26a, miR-181a, and miR-125b were also abundantly detected and, together with let-7 (a-i) family members, made up >65% of all identified miRNAs (Table S2, S3). No viral miRNAs were detected in any of the samples analyzed.

**Figure 1:**
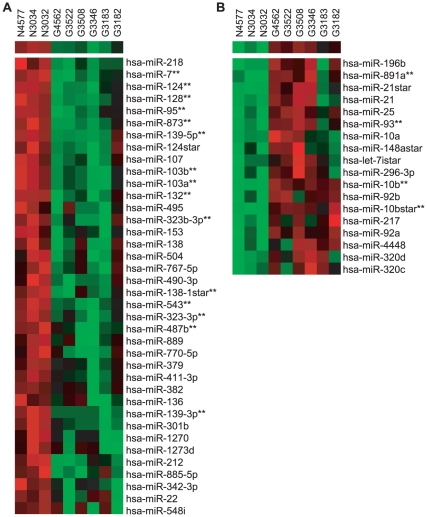
Differentially expressed miRNAs in glioblastomas versus non-tumor brain tissue. miRNA expression levels were determined by deep sequencing. Shown are heat maps for the 38 miRNAs most significantly down-regulated (A) and the 18 miRNAs most significantly up-regulated (B) in the six glioblastoma (G) samples compared to non-tumor brain tissue (N) (mean fold change >4, p<0.05 by t test). Highlighted are miRNAs that fulfill the FDR-adjusted selection criteria (** p<0.1) as reported in Table S6. Two additional miRNAs, miR-let-7d and miR-598, also meet the FDR cut-off but exhibit a fold change <4 and are not shown. Prior to hierarchical clustering, deep sequencing reads with RPKM values >1 as determined by miRNAkey [32] were log-transformed, and standardized across all samples to a mean of 0 and standard deviation of 1. Clustering was performed using Pearson's correlation (average linkage method) as the distance matrix.

To determine differences in miRNA expression patterns in glioblastomas versus non-tumor brain tissue, we used the miRNAkey pipeline, implementing the SeqEM option to account for reads mapping to multiple miRNAs [32]. 449 mature miRNAs and miRNAs* were identified using a filter of RPKM score >1 to normalize read counts between different libraries and by considering only those miRNAs occurring in at least two out of three non-tumor brain libraries or 50% of the glioblastoma libraries (Table S4). From this set of 449, 68 miRNAs and miRNA*s were differentially expressed between non-tumor brain tissues and glioblastomas (p<0.05, Student's t test; Table S6). When we applied the standard FDR-adjusted p-value <0.05 cut-off, only nine miRNAs were identified that met the criteria (Table S5). Given the much broader dynamic range of deep sequencing compared to finite platforms such as miRNA microarrays or real-time PCR arrays, we therefore used a FDR cut-off of p<0.1. 21 aberrantly expressed miRNAs met the criteria (Fig. 1, Table S5, Table S6, Fig. S2).

Significantly up-regulated miRNAs in glioblastoma samples included miR-10b and miR-93 (Table S6). We also observed high read counts for miR-21, amongst other miRNAs, in all glioblastoma samples compared to non-tumor samples, congruent with previous studies showing that this miRNA is highly up-regulated in glioblastoma (Table S4) [5], [9], [14], [15]; however, there was variability in the values for miR-21 across the samples and thus, the miRNA did not meet the final FDR selection criteria.

17 of the 21 significantly dysregulated miRNAs were down-regulated in glioblastomas (FDR-adjusted p-value <0.1, fold change >3) including miR-124, miR-95, miR-132, miR-139-5p, miR-7, miR-128, miR-487b, and miR-873 (Fig. 1A, Table S6). miR-212, miR-218, and miR-379, amongst others, also exhibited lower read counts in glioblastoma compared to non-tumor brain tissue (Table S4), but did not meet the final FDR selection criteria. While the aberrant expression of many of these miRNAs is consistent with previous profiling studies (Table S6) [9], [14]–[16], [33], deep sequencing analysis further revealed at least four significantly down-regulated miRNAs (miR-95, miR-543. miR-598, and miR-873) that were not previously reported to be dysregulated in brain cancers (Table S6).

### Validation of miRNA expression patterns by primer extension

**Figure 2:**
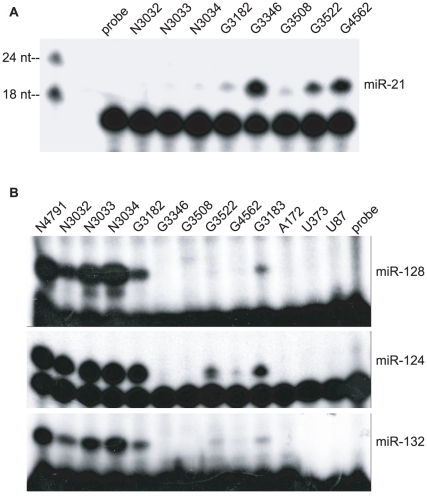
MiRNA expression in brain tissue samples and glioblastoma cell lines. A. miR-21 expression detected by primer extension. B. miR-128, miR-124, and miR-132 expression levels detected by primer extension. N  =  non-tumor brain tissue. G  =  glioblastoma. A172, U373, and U87 are glioblastoma cell lines. 6 µg total RNA were used for each reaction. Free probe is shown as a negative control for each miRNA assayed. Primer extension reactions were resolved on 15% TBE-Urea polyacrylamide gels and exposed to film for either 1 hr (miR-21) or overnight (miR-128, miR-124, miR-132).

To validate results from our deep sequencing experiments, we selected four differentially expressed miRNAs for further analysis: miR-21, miR-128, miR-124, and miR-132. Primer extension analysis confirmed enhanced expression of miR-21 (Fig. 2A) and decreased expression of miR-128, miR-124, and miR-132 (Fig. 2B) in glioblastomas compared to non-tumor brain tissue samples. Additionally, we could not detect expression of miR-128, miR-124, or miR-132 in three glioblastoma cell lines (A172, U373, and U87).

### Variations in 5p and 3p strand dominance

MiRNAs are derived from either the 5p or 3p arm of a ∼60 nt hairpin precursor termed a pre-miRNA. Dicer cleaves the pre-miRNA hairpin into a ∼22 nt dsRNA duplex, and one strand of the duplex is incorporated into the Argonaute-containing RNA-induced silencing complex (RISC) to become the mature miRNA. The passenger strand, termed miRNA*, is then degraded. One factor influencing strand selection is the thermodynamic stability of the miRNA duplex termini [34]. In some instances, such as when the termini of the duplex exhibit similar stabilities, either strand of the duplex can become a mature miRNA, resulting in RISCs that are loaded with a functional miRNA* strand [13].

We detected a number of miRNAs processed from both the 5p and 3p arms of their respective pre-miRNAs, such as miR-136, miR-151, miR-140, and miR-330 (Table S2, S3). To determine whether there were any differences in miRNA strand selection in glioblastomas versus non-tumor brain tissue, we compared 5p:3p ratios for those miRNAs falling into this category. In at least 70 instances, we observed a switch in the dominant arm of the pre-miRNA annotated for a given miRNA in at least one of the nine samples (data not shown). Notably, three miRNAs exhibited strand selection preferences dependent on the sample origin. The 3p miRNAs for miR-330 and miR-204 were preferentially detected in non-tumor brain tissue, while the 5p miRNAs were preferentially detected in glioblastomas (Table S7; Fig. S3B). Similarly, miR-30a-5p was enriched in glioblastomas while in non-tumor brain tissue, miR-30a-5p and miR-30a-3p were expressed at similar levels (Table S7; Fig. S3B). Such changes may indicate post-transcriptional processing differences—for instance, the absence or presence of a factor in glial versus non-glial cells that perturbs miRNA strand selection.

### miRNA editing and miRNA sequence variants in the brain

MiRNA dysregulation can occur at many levels. Genomic mutations in miRNA loci may affect expression and/or processing, while epigenetic silencing, chromatin modifications, and/or altered levels of transcription factors may affect expression of the primary miRNA transcript. Additionally, single nucleotide changes have been shown to affect miRNA expression, processing, and/or function [35]. One post-transcriptional miRNA modification is enzymatic editing of the dsRNA primary miRNA transcript by ADAR (adenosine deaminase acting on RNA) which results in the conversion of an adenosine (A) to an inosine (I) [24]. A to I conversions can influence loading of miRNAs into RISC [34] and also alter the targeting capacity of a miRNA, especially if the editing occurs within the miRNA seed region [24], [25]. A single A to I conversion in the seed of miR-376a-5p, for example, redirects the edited miRNA to a new set of mRNA targets [25]. In deep sequencing libraries, this conversion is marked by the substitution of A with G.

To determine whether any miRNAs were post-transcriptionally edited, we examined miRNA seed sequences (nt 2–7) for A to G substitutions. Congruent with previous studies, A to G changes occurred in >60% of the reads for members of the miR-376 family (miR-376a, b, and c) at nucleotide position six of the mature miRNA sequence (Table 1, Fig. S3A) [36]. We also noted A to G substitutions at nucleotide position four for miR-381 and position five for miR-411, miR-200b, and miR-379 (Table 1, Fig. S3A). A to G substitutions also occurred in the seed region of miR-320 family members; however, these miRNAs were unusual in that several positions (two, three, and four) exhibited nucleotide substitutions (Fig. S3A and data not shown). A to G substitutions for each of the miRNAs were found in both the glioblastoma and non-tumor brain tissue libraries, and there were no significant differences in the averaged abundance of edited miRNAs between the two sample types (Table 1). Furthermore, several of these miRNA variants (miR-376 family members, miR-411, miR-379, and miR-320a) have previously been described in deep sequencing studies performed on human brain [36], [37], and therefore, are not specific to glioblastoma.

**Table 1:** A to G substitutions in miRNA seed sequences.

	Average % Edited		
miRNA	NB	GBM	Sequence
miR-376b	92%	91%	AUCAU**A**GAGGAAAAUCCAUGU
miR-376c	63%	73%	AACAU**A**GAGGAAAUUCCACGU
miR-376a	62%	69%	AUCAU**A**GAGGAAAAUCCACGU
miR-381	30%	38%	UAU**A**CAAGGGCAAGCUCUCUGU
miR-411-5p	21%	20%	UAGU**A**GACCGUAUAGCGUACG
miR-200b	12%	7.3%	UAAU**A**CUGCCUGGUAAUGAUGAC
miR-379	7.5%	5.2%	UGGU**A**GACUAUGGAACGUAGG

No significant differences were observed in the occurrence of A to I editing in glioblastoma (GBM) versus non-tumor brain (NB) samples. Edited “A”s are in bold.

MiRNA sequence variations can also arise due to processing differences at the 5′ and 3′ ends. While 3′ end heterogeneity is commonly observed for miRNAs, 5′ end variations are less common and can have dramatic consequences on miRNA targeting since a single 5′ nucleotide addition or truncation alters the sequence of the miRNA seed [27]. To determine whether 5′ end variations in miRNA sequences might be associated with the aberrant expression of specific miRNAs, we examined the major sequence variants for all identified miRNAs.

We identified 29 miRNAs that exhibited 5′ nucleotide truncations or additions in at least 10% of the mapped reads (Table S8). Several of the miRNAs down-regulated in glioblastoma (including miR-124-3p and miR-323-3p) exhibited 5′ truncations or additions while one up-regulated miRNA, miR-10b-5p, exhibited 5′ variations (Table S8). These 5′ nucleotide variations were observed in all nine sequencing libraries, and with the exception of miR-124-3p, there was no correlation of 5′ end variability and miRNA expression differences in tumors versus non-tumor samples (Table S4, S7, and S8). We did, however, observe major differences in the dominant isoform for at least six miRNAs when comparing glioblastomas versus non-tumor samples (Table S7). Interestingly, the dominant form of miR-324-3p was consistent with the miRNA sequence reported in miRBase; however, in non-tumor brain, we observed a significant number of reads mapping to a miR-324-3p isoform that was two nucleotides longer at the 5′ end (Table S7). miR-324, together with four other miRNAs that show isoform differences in tumor versus non-tumor samples (miR-106b, miR-335, and miR-411), also exhibited variations in the major arm of the pre-miRNA expressed as a miRNA (Table S2, S3, S4, S6). In fact, one third of the miRNAs with 5′ end polymorphisms showed variations in strand selection preference (Table S4, S6, and data not shown).

Two miRNAs worth noting are miR-30a and miR-330. Two major isoforms were observed for both miR-30a-3p and miR-330-3p. 5′ end variations occurred at the same frequency in glioblastomas and non-tumor brain samples for both these miRNAs (Table S7); however, in the majority of the glioblastoma libraries, the miRNAs expressed from the 3p arms were under-represented while miR-30a-5p and miR-330-5p were enriched (Fig. S3B). These data suggest that 5′ end polymorphisms can reflect fluctuations in strand dominance and presumably, reflect variations in Drosha processing of the primary miRNA transcript.

### miRNAs as potential therapeutic tools to regulate cell suicide gene expression

After comprehensively determining which miRNAs are dysregulated in glioblastoma, our next objective was to use this information in the practical design of a miRNA-regulated gene therapy vector expressing a cell suicide gene, namely, herpes simplex virus 1 thymidine kinase (HSV-TK). Cells expressing HSV-TK selectively undergo apoptosis when cultured in the presence of the deoxyguanosine analogue ganciclovir (GCV) [38]. HSV-TK-based lentiviral vectors are currently in phase I/II clinical trials for glioblastoma treatment [39], [40]. While lentiviral vectors are ideal gene therapy tools as they can be pseudotyped to enter a variety of cell types, one major drawback in glioblastoma treatment is that such vectors can also enter the tissue surrounding the tumor, killing otherwise healthy cells, which could have dire consequences in the brain. We hypothesized that incorporation of artificial miRNA target sites into the HSV-TK 3′UTR could be an effective means of restricting cell suicide gene expression to tumor cells.

miR-128 is one of the most significantly and consistently down-regulated miRNAs in glioblastomas and glioblastoma cell lines according to data presented here (Fig. 1) as well as previous studies [9], [14], [15], [31]. Our sequencing data reveal that miR-128 has an invariant sequence and does not exhibit post-transcriptional editing or significant 5′ end variation. Additionally, miR-128 is highly enriched in the brain as it is expressed by neurons and is induced during neuronal differentiation of embryonal carcinoma (EC) cells [20], [21], thus making it an ideal candidate to limit cell suicide gene expression. We engineered two miR-128 target sites into the HSV-TK 3′UTR (HSV-TK-128.2X) and then transiently expressed the cell suicide gene in glioblastoma cell lines.

**Figure 3:**
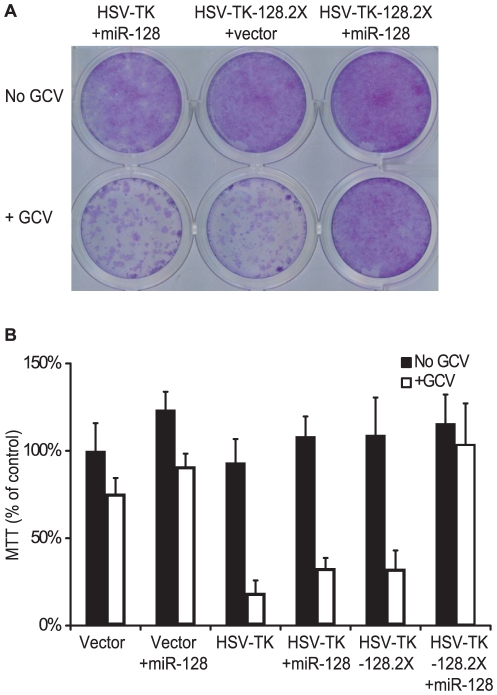
MiRNA regulation of HSV-TK in U373 glioblastoma cells. A. Crystal violet staining of U373 glioblastoma cells expressing HSV-TK or HSV-TK-128.2X following GCV treatment. pcDNA3-HSV-TK-128.2X contains two perfect binding sites for miR-128 in the 3′UTR of HSV-TK. Cells were co-transfected with either pcDNA3 (+vector) or pcDNA3-miR-128 (+miR-128) and subjected to 10 µg/mL ganciclovir selection (+GCV) for 10 days prior to staining. B. miR-128 rescues HSV-TK-128.2X expressing U373 cells from GCV-induced cell death. U373 cells were co-transfected with pcDNA3 (vector), pcDNA3-HSV-TK, or pcDNA3-HSV-TK-128.2X and either pcDNA3 as control or pcDNA3-miR-128 (+miR-128). Cells were plated in 24-well plates with media containing 10 µg/ml GCV and cell viability was measured at 10 days by MTT assay as described in Methods (n = 3). Absorbance values are normalized to control cells (transfected with empty vector and grown in media without GCV).

A172 and U373 glioblastoma cells were transfected with pcDNA3-HSV-TK-128.2X and cultured in media containing GCV. To determine the lowest effective dose for GCV selection, cells were plated at 2×10^4^ cells per well in 24-well plates and incubated for eight days in media containing various concentrations of GCV (0, 0.1, 1, 10, 100, and 1000 µg/ml) (Fig. S5). A dose of 10 µg/ml GCV was chosen for subsequent transfection experiments (Fig. 3, S5B & C).

To specifically demonstrate miRNA regulation of HSV-TK-128.2X, we cloned a ∼250 nt DNA fragment of the region encompassing pre-miR-128-1 into pcDNA3. miR-128 expression was confirmed from this vector using luciferase indicator assays (Fig. S4). Next, U373 cells, which do not express detectable levels of miR-128 (Fig. 2, 5), were co-transfected with pcDNA3-HSV-TK-128.2X and the miR-128 expression vector. Ectopic expression of miR-128 in glioblastoma cells inhibited HSV-TK-128.2X-mediated cell death when cells were cultured in the presence of GCV as shown by trypan blue exclusion (Fig. S4), crystal violet staining, and MTT viability assays (Fig. 3). In contrast, miR-128 did not rescue the viability of U373 cells transfected with an analogous HSV-TK vector lacking the miR-128 binding sites (Fig. 3). Interestingly, the growth and viability of both A172 and U373 cells was not significantly affected by the overexpression of miR-128 (Fig. 3B, S5), contrary to previous studies using other glioma cell types that may retain more stem-cell like characteristics [14], [41].

### Endogenous miR-128 protects neuronal cells from HSV-TK induced cell death

**Figure 4:**
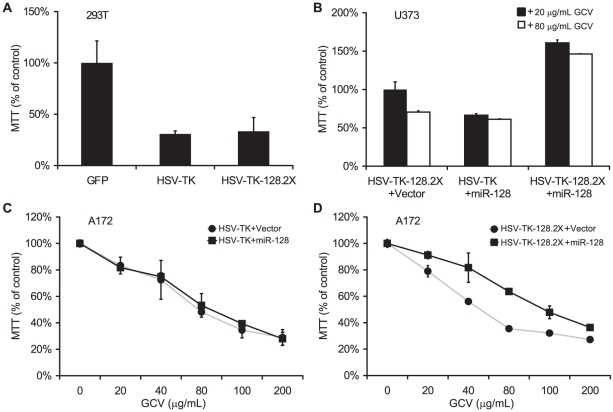
MiRNA regulation of HSV-TK in transduced glioblastoma cell lines. A. miR-128 binding sites have no effect on HSV-TK-mediated killing in cells lacking miR-128. 293T cells were transduced with pLCE (GFP), pL-HSV-TK, or pL-HSV-TK-128.2X and cultured for two weeks in the presence of 40 µg/ml GCV (n = 2). Cell viability was measured by MTT assay. B. miR-128 rescues glioblastoma cells from killing by HSV-TK-128.2X. U373 cells were transduced with indicated vectors in 6-well plates. 24 hrs post-transduction, cells were transfected with pcDNA3 or pcDNA3-miR-128 using Lipofectamine 2000. 24 hrs later, cells were plated in 24-well plates and cultured for 2 weeks in 20 µg/ml or 80 µg/ml GCV (n = 2). C and D. Dose-dependent response to GCV. A172 cells were transduced with indicated vectors, then transfected with pcDNA3 (grey lines, circles) or pcDNA3-miR-128 (black lines, squares). 24 hrs later, cells were plated in 24-well plates and cultured for 2 weeks in the presence of GCV (n = 2). Cell viability was measured by MTT assay (B–D).

We next tested miRNA regulation of HSV-TK-128.2X in cell lines stably expressing HSV-TK. 293T, U373, and A172 cells were transduced with pLCE (GFP), pLC-HSV-TK, or pLC-HSV-TK-128.2X and then transfected with a miR-128 expression plasmid or empty vector as indicated (Fig. 4). HSV-TK-mediated killing was unaffected by the presence of the miR-128 target sites in the absence of miR-128 (Fig. 4A, S5B). Additionally, overexpression of miR-128 did not affect HSV-TK-mediated killing in the absence of miR-128 target sites (Fig. 4B & C). We tested A172 cells with increasing concentrations of GCV. At the highest dose, 200 µg/mL GCV, ∼70% of HSV-TK-expressing cells were killed (Fig. 4C & D). Similar to Fig. 3, introduction of a miR-128 expression plasmid rescued both U373 and A172 cells from cell death when the miR-128 binding sites were present in the HSV-TK 3′UTR (Fig. 4B–D).

**Figure 5:**
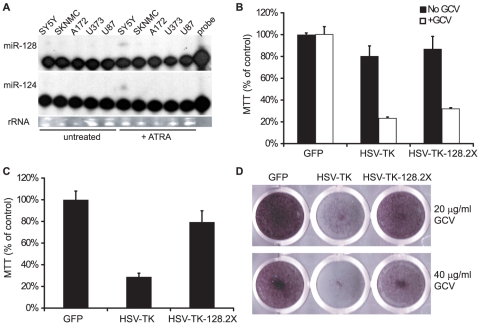
Endogenous miRNA regulation of HSV-TK protects cells from ganciclovir-induced cell death. A. Brain-enriched miRNAs, miR-128 and miR-124, are induced during neuronal differentiation of SH-SY5Y cells. Cells were grown in the presence of 10 µM ATRA for >4 days. 10 µg total RNA were used for primer extensions to detect miRNA expression. SH-SY5Y and SKNMC are neuroblastoma cell lines. A172, U373, and U87 are glioblastoma cell lines. B. SH-SY5Y cells were transduced with lentiviral vectors expressing GFP, HSV-TK, or HSV-TK-128.2X, plated in 24-well plates at 1×10^4^ cells per well, and treated with or without 40 µg/ml GCV for 2 weeks (n = 2). C and D. Endogenous miR-128 restricts the activity of HSV-TK-128.2X. SH-SY5Y cells were transduced as in (B) and plated in 24-well plates at 2.5×10^4^ cells per well. Cells were cultured in the presence of 10 µM ATRA to induce neuronal differentiation and 40 µg/ml GCV for two weeks (n = 2). Cell viability was measured by MTT assay (C), and crystal violet staining of SH-SY5Y cells treated with either 20 µg/ml or 40 µg/ml GCV is shown in (D).

Finally, to determine whether endogenous brain-specific miRNAs could protect cells from a miRNA-regulated cell suicide gene, we tested the human neuroblastoma cell line, SH-SY5Y. When cultured in the presence of all-trans retinoic acid (ATRA), SH-SY5Y cells undergo morphological changes, differentiate into neuron-like cells, express neuronal markers, and up-regulate a number of brain-specific miRNAs [42], [43]. We treated two neuroblastoma cell lines with 10 µM ATRA for five days and assayed cellular miRNA expression by primer extension (Fig. 5A). Three glioblastoma cell lines were also treated with ATRA as controls. Congruent with previous studies [42], [43], ATRA treatment of SH-SY5Y cells induced expression of the brain-enriched miRNAs miR-128 and miR-124 (Fig. 5A) as well as miR-132 and miR-7 (not shown).

SH-SY5Y cells were then transduced with pLCE (GFP), pLC-HSV-TK, or pLC-HSV-TK-128.2X and cultured in media with or without GCV (Fig. 5B). HSV-TK-mediated killing of undifferentiated SH-SY5Y cells was again unaffected by the presence of the miR-128 binding sites. However, when SH-SY5Y cells were chemically induced to undergo neuronal differentiation, cells transduced with HSV-TK-128.2X were protected from cell death as assayed by MTT and crystal violet staining (Fig. 5C and D). Thus, the expression of endogenous, brain-enriched miR-128 can restrict the effects of a cell suicide gene containing cognate miRNA binding sites.

## Discussion

Here, we present a comprehensive overview of the repertoire of miRNAs expressed in glioblastoma samples, and we identify several miRNAs that are dysregulated in glioblastoma. Additionally, we report a number of miRNA sequence variants that are expressed in the brain. MiRNA regulation of gene expression is an important aspect of tumorigenesis and cancer progression, and gaining a better understanding of the miRNAs that are dysregulated in brain cancers therefore has the potential to reveal mechanistic insights into the transformed state as well as open the door to new treatment modalities.

Our findings from deep sequencing of brain tissue and tumor samples significantly add to the list of miRNAs that are aberrantly expressed in brain tumors. We identified several new miRNAs that were not previously reported to be dysregulated in glioblastomas, including miR-95, miR-543, miR-598, and miR-873 (Fig. 1, Table S6). Consistent with other glioma miRNA profiling studies [9], [14], [15], [31], we also observed down-regulation of miR-124, miR-128, miR-132, and miR-7, and up-regulation of miR-10b, amongst others, demonstrating that high-throughput sequencing can be an effective method for profiling miRNA expression.

Compared to microarray and PCR-based profiling platforms, which detect a pre-determined set of miRNAs, high-throughput sequencing provides a more complete picture of the miRNAs expressed in a given sample and is not restricted in the number of individual miRNAs that can be detected. While these advantages of high-throughput sequencing allow for a greater dynamic profiling range, the method is not without limits. Using the standard FDR selection criterium of p<0.05, only nine miRNAs were identified here as significantly dysregulated in glioblastoma samples compared to non-tumor brain tissue (Table S5). Based on the read counts for individual miRNAs in Tables S2, S3, and S4, previous profiling studies (listed in Table S6), and data shown here regarding miRNAs such as miR-21 and miR-128 (Fig. 2, Fig. 5) that do not meet the standard FDR-adjusted p<0.05 criteria (Table S5), clearly additional miRNAs identified here are dysregulated in glioblastoma (Fig. 1, Tables S4 and S6). We therefore expanded our cut-off to p<0.1 to include 21 significantly dysregulated miRNAs, 17 of which were down-regulated in glioblastoma.

Importantly, high-throughput sequencing allowed us to analyze sequence variations in the detected miRNAs and investigate the possible presence of exogenous miRNAs such as viral miRNAs. In this study, we observed significant levels of A to I editing for seven cellular miRNAs expressed in the brain and 5′ end polymorphisms for at least 30 miRNAs, which could have dramatic consequences on mRNA targeting [25], [27]. In particular, 5′ end sequence heterogeneity may have functional implications as there are observable differences in the dominant isoforms for a few miRNAs (miR-335, miR-106b, miR-324) expressed in glioblastomas.

One question we were able to address by deep sequencing was whether any viral miRNAs might be expressed in the tumors and perhaps, might play a role in pathogenesis. Human cytomegalovirus (HCMV) and other neurotropic viruses, such as the polyomavirus JC virus (JCV), which replicates in glial cells, have recently been implicated in gliomas, tumors of the central nervous system, and other brain cancers [44]–[49]. Although it is unclear at this point whether these viruses have any specific role in tumorigenesis, the incidence of HCMV infection in patients with glioblastoma multiforme is high; viral proteins and viral DNA have been detected in over 80% of glioblastomas [46], [47]. Both HCMV and JCV are known to express high levels of viral miRNAs during infection [50]. Therefore, we aligned sequencing reads from all samples to known viral miRNAs present in miRBASE v 16.0 as well as the genomes of human α-, β- and γ-herpesviruses (HSV-1, HSV-2, HCMV, HHV-6A/B, HHV-7, EBV, KSHV) and neurotropic polyomaviruses (JCV and SV40). No viral small RNAs were detected in any of the nine samples analyzed (data not shown), indicating that viral miRNAs are likely not a contributing factor to the development or progression of glioblastoma.

It is hypothesized that glioblastomas, like other cancers such as leukemia [51], arise and are maintained by a population of progenitor cells with stem-like characteristics including the ability to self-renew for unlimited proliferation [21], [52], [53]. Indeed, the glioblastoma miRNA signature supports this hypothesis. Brain-enriched miR-124 and miR-7, for example, show poor expression in neural progenitors, but are highly expressed in neurons and have been implicated in neural differentiation [20], [23], [54]. Similarly, CREB-induced miR-132 and miR-212, which share seed sequence homology, promote dendritic outgrowth from newborn neurons [55] and can target methyl CpG binding protein 2 (MeCP2), a regulator of neuroplasticity [56], [57]. Finally, miR-128, which is also abundantly expressed in neurons, has been shown to target the Bmi-1 stem cell renewal factor [14]. miR-7, miR-124, miR-128, miR-132, and miR-212 are amongst the most highly down-regulated miRNAs found in glioblastomas compared to non-transformed cells (Fig. 1, Table S4, S6). Furthermore, miR-7, miR-124, and miR-128 have been reported to impair cell growth and proliferation when over-expressed in glioma-derived stem cells [14], [15], [19], [41].

At least 20 cellular miRNAs were differentially expressed in the six glioblastomas assayed here compared to non-tumor brain tissue, many of which (miR-128, miR-124, miR-7, miR-132, miR-139) are consistently dysregulated in not only gliomas but also other brain cancers including medulloblastomas and neuroblastomas [33]. It is conceivable that several of the down-regulated miRNAs with no defined functions at this point (i.e., miR-873, miR-95, miR-543) may exhibit tumor suppressor activity and normally target components of key signaling pathways that promote and maintain the growth and survival of glioma cells as has recently been reported for miR-10b [17].

While elucidating the roles of miRNAs in tumor formation and progression is certainly an important area of research, we took a different approach here and used our miRNA profiling data to explore the possible use of miRNAs in regulating cell suicide gene therapy. The use of tissue-specific miRNAs to regulate transgene expression for therapeutic applications has been investigated in multiple cell types, including lymphoid and myeloid cells, muscle cells, liver cells, and brain cells [28]–[31]. One study done with brain developed an insect baculovirus-based vector expressing HSV-TK under the regulation of three miRNAs (miR-31, miR-127, and miR-143) reported to be under-expressed in glioblastoma cell lines [31]. This vector showed the same therapeutic efficacy in glioblastoma U87 cells as a control HSV-TK vector lacking artificial miRNA target sites, but when introduced into human astrocytes, which express the three selected miRNAs, showed restricted HSV-TK expression, leading to an increase in cell viability. In our analysis of primary human glioblastoma tissue samples and brain tissues, miR-31 was not detectable, miR-143 was down-regulated in only half of the glioblastoma samples analyzed, and miR-127 exhibited only modest down-regulation in glioblastomas (Table S4). Instead, we focused on miRNAs that are consistently down-regulated in glioblastomas [9], [14], [15] (Fig. 1A, Table S6), and chose miR-128 as the best candidate to restrict cell suicide gene expression. While miRNA effects on target mRNAs are usually modest, using a miRNA such as miR-128, which is brain-enriched and highly expressed in non-glioma cells, potentially maximizes the efficacy of miRNA regulation. Furthermore, reduced expression of miR-128 is not specific to just glioblastomas as this miRNA is also down-regulated in other brain cancers [33]. Using an in vitro model to recapitulate expression of brain-enriched miRNAs, we demonstrated that differentiated neuronal SH-SY5Y cells transduced with a miR-128-regulated HSV-TK lentiviral vector are indeed resistant to killing by GCV due to the expression of endogenous miR-128.

In conclusion, the data presented here provide a comprehensive analysis of the miRNA signature in glioblastoma and demonstrate the potential application of miRNA-regulated genes in the therapeutic treatment of brain cancers— an approach that could be readily extended to cancers of other organs.

## Materials and Methods

### Cell lines and tissue samples

U373MG, A172, and U87MG glioblastoma cell lines were obtained from the Duke Cell Culture Repository and maintained in Dulbecco's modified Eagle's medium (DMEM) supplemented with 10% fetal bovine serum (FBS), 0.1 mM MEM non-essential amino acids, 1 mM sodium pyruvate, and antibiotics. 293T cells were maintained in DMEM supplemented with 10% FBS and antibiotics. SH-SY5Y and SKNMC neuroblastoma cell lines were maintained in RPMI 1640 supplemented with 10% FBS and antibiotics. To induce neuronal miRNA expression, 10 µM all-trans retinoic acid (ATRA) (Sigma) was added to cells for a minimum of four days.

Human brain tissue samples were obtained through the Brain Tumor Center Biorepository of the Preston Robert Tisch Brain Tumor Center at Duke. De-identified tissues were used for all studies and the data were analyzed anonymously. Non-tumor brain tissue samples were taken from the right, temporal lobes of patients with cortical dysplasia. Table S1 describes the glioblastoma samples. All tissue samples were obtained fresh, flash frozen in Tissue-Tek OCT, and stored at -80°C prior to RNA extraction. None of the samples were post-mortem.

### Deep Sequencing and Bioinformatics

Small RNA libraries were prepared as previously described [58]. Briefly, RNA was extracted from OCT-embedded tissues using TRIzol and lysis matrix A (1.4 mm ceramic) (Mo Bio Laboratories, Inc., CA) with a homogenizer for 10 sec. 30 µg of total RNA was size-fractionated on 15% TBE-UREA polyacrylamide gels. Small RNAs (18–24 nt) were extracted and ligated to adapter sequences for Illumina sequencing. Each 5′ adapter contained a five nucleotide molecular barcode (Table S3). Samples were combined and sequenced over four lanes on an Illumina GA2 sequence analyzer at the Duke IGSP Core Sequencing Facility.

Sequences (36 nt in length) were obtained in FASTA format. Raw sequencing reads were trimmed for adapter sequences and filtered for size (>16 nt) using the FASTX toolkit (http://hannonlab.cshl.edu/fastx_toolkit). FASTA files containing all unique sequences for the nine libraries are provided (Supporting Information files S1, S2, S3, S4, S5, S6, S7, S8, and S9). For differential expression analysis, sequences were aligned to human and viral miRNAs/miRNA*s present in miRBASE v 16.0 (allowing 2 mismatches) using the miRNAkey pipeline and the SeqEM option to enable analysis of reads mapping to multiple miRNAs [32]. miRNAs with a RPKM scores >1.0 were kept for further analysis (Table S4). For cluster analysis, RPKM normalized read counts were log-transformed, filtered by t-score (p<0.05), and standardized across all samples to have the mean equal to zero and the standard deviation (SD) ±1. Clustering of miRNAs exhibiting mean fold change >4.0 was performed in Cluster 3.0 using the average linkage method and Pearson's correlation and visualized in TreeView [59]. False discovery rate (FDR)-adjusted p-values were calculated using MicroSoft Excel and are listed in Table S5 for all detected miRNAs.

To interrogate miRNA sequence variations including A to I editing, reads were aligned to human pre-miRNAs using NCBI BLAST with the following parameters (W = 15, D = 2, p = 90). For editing, reads were also perfectly aligned to in silico A to G substituted mature miRNA/miRNA* sequences. Reads were parsed further using scripts from the miRDEEP pipeline [60] and imported into Microsoft Excel.

### Primer Extension to detect mature miRNAs

Primer extensions were performed with 5 µg of total RNA using the AMV PE kit according to the manufacturer's protocol (Promega). Oligonucleotides used for probes are listed (Table S9) and were end-labeled using γ^32^P-ATP and T4 polynucleotide kinase. To detect individual miRNAs, a master mix was prepared for each probe and divided equally amongst the reactions. Reverse transcription products were separated on 15% TBE-urea polyacrylamide gels and exposed to film.

### Plasmid constructs, transfections, and lentiviral transductions

A ∼250 nt region encompassing pre-miR-128-1 was PCR amplified from human genomic DNA and cloned into pcDNA3 downstream of the CMV promoter using *XhoI* to *XbaI* sites. The miR-128 indicator vector (pcDNA3-GL3-128.2X) contains a firefly luciferase cassette and two fully complementary binding sites for miR-128 inserted into the 3′UTR using *XhoI* to *XbaI* (Fig. S4). pcDNA3-HSV-TK was generated by amplifying the cell suicide gene, HSV-TK [61], and cloning into pcDNA3 using *HindIII* to *EcoRI.* pL-CMV-eGFP (pLCE) has been previously described [62]. To generate pL-HSV-TK, eGFP was replaced with PCR-amplified HSV-TK *NheI* to *XbaI.* Two miR-128 binding sites were then inserted into the 3′UTR *XhoI* to *XbaI.* Oligonucleotides are listed in Table S9.

Glioblastoma cell lines were transfected using Lipofectamine 2000 according to manufacturer's instructions (Invitrogen). For packaging lentivirus vectors, 293T cells were plated in 10 cm plates and co-transfected with 5 µg pL-based vector and 1.25 µg each pMDL-gpRRE, pRSV-REV, and pVSVg [62] using the calcium phosphate transfection method. Lentivirus was filtered from the supernatant 48 hrs post-transfection and used for transductions.

### Crystal Violet Staining and MTT assays

Transfections and transductions were carried out in 6-well plates. 24 hrs post-transfection or post-transduction, cells were seeded in 24-well plates at 15,000 cells per well, or as indicated, and placed under ganciclovir (GCV) selection for 10–14 days. SH-SY5Y cells were seeded in 24-well plates at 25,000 cells per well 48 hrs post-transduction and cultured for 10 days in media containing GCV and either 10 µM ATRA or 10 µM di-methyl sulfoxide (DMSO) vehicle control.

For crystal violet staining 10–14 days post-treatment with GCV, cells were washed twice with phosphate buffered saline (PBS) and fixed at room temperature for 15 min. in 30% methanol in PBS containing 0.1% crystal violet. Wells were de-stained with 30% methanol in PBS lacking crystal violet. To assay cell proliferation 10–14 days post-treatment, cells were washed with PBS and the media replaced with 1∶1 mixture of OptiMEM without phenol red (Gibco) supplemented with 10% FBS and MTT (3-(4,5-dimethylthiazol-2-yl)-2,5-diphenyltetrazolium bromide) reagent (5 mg/ml MTT in PBS) (Invitrogen). Cells were incubated for 2 hrs at 37°C, lysed in buffer (isopropanol containing 0.5% NP40 and 4 mM HCl), and incubated for an additional 2 hrs at 37°C prior to analysis. Lysates were read in 96-well microtiter plates using a plate reader (Absorbance  = 562 nm), and relative cell growth was determined by comparing absorbance values of treated cells expressing HSV-TK to absorbance values of control cells (treated cells transfected with empty vector or expressing eGFP).

## Supporting Information

File S1 Files S1–S9 are FASTA files of deep sequencing reads for each of the nine samples listed in Table S1. Reads have been stripped of barcodes and are in the format: >ID_UniqueReadNumber_xReadCount, SEQUENCE.
(FASTA)

File S2 (FASTA)

File S3 (FASTA)

File S4 (FASTA)

File S5 (FASTA)

File S6 (FASTA)

File S7 (FASTA)

File S8 (FASTA)

File S9 (FASTA)

Table S1 **Sequenced brain samples.**
(DOC)

Table S2 **Glioblastoma miRNA transcriptome.**
(XLSX)

Table S3 **Cortical dysplasia brain miRNA transcriptome.**
(XLSX)

Table S4 **RPKM scores for nine deep sequencing libraries.**
(XLSX)

Table S5 **p-values and False Discovery Rate (FDR)-adjusted p values for all miRNAs detected in glioblastomas and non-tumor brain tissues.**
(XLSX)

Table S6 **Differentially expressed miRNAs in glioblastomas (GBM) vs non-tumor brain (NB).**
(XLSX)

Table S7 **5′-end variations and strand dominance in select miRNAs.**
(XLSX)

Table S8 **5′-end variations alter miRNA seed sequences.**
(XLSX)

Table S9 **Oligonucleotides used in this study for deep sequencing, primer extension, and cloning.**
(DOC)

Figure S1 **Deep sequencing libraries.** A and B. The read length distributions for each deep sequencing library are shown. The majority of reads in each library are 22-23 nt in length, which is the average length of a mature miRNA.
(EPS)

Figure S2 **Differentially expressed miRNAs.** Scatter-plot drawn in ‘R’ of mean RPKM scores (miRNA expression values) for glioblastoma miRNAs (x-axis) compared to non-tumor brain miRNAs (y-axis). Each point represents a unique miRNA. Differentially expressed miRNAs (p<0.05) are highlighted in red. Points that fall above the black line are indicative of miRNAs that are down-regulated in glioblastomas. The linear regression of the entire sample set is shown in blue.
(EPS)

Figure S3 **Variations in miRNA sequences.** A. Post-transcriptional editing occurs in the seed regions of several brain-enriched miRNAs. Shown is the average percent of A to I editing in seed regions (nt 2-7) for individual miRNAs in all nine samples analyzed. This value was determined by comparing the number of reads exhibiting A to G substitutions at a specific nucleotide position for a given miRNA to the number of total reads mapping to the miRNA. All miRNAs identified across the nine deep sequencing libraries were interrogated. Each blue dot represents one individual miRNA, which are ordered based on the amount of editing events (x-axis). The top nine miRNAs exhibiting significant A to I editing are highlighted. B. miR-330, miR-30a, and miR-204 show differences in the 5p:3p ratios in glioblastomas (GBM) compared to non-tumor brain (NB). p values are reported according to Welch's t test.
(EPS)

Figure S4 **Ectopic expression of miR-128.** A. 293T cells were co-transfected with 1 µg pcDNA3 (vector) or pcDNA3-miR-128 (miR-128), 50 ng firefly luciferase expression vector, pFLuc-128.2x containing two miR-128 binding sites or pFLuc-2X control, and 10 ng renilla luciferase expression vector, pRLuc, as internal control using Lipofectamine 2000 (Invitrogen). Lysates were harvested and luciferase activity measured 72 hrs post-transfection using the dual luciferase assay kit (Promega) (n = 3). B. A172 or U373 glioblastoma cells were transfected as in (A). Luciferase activity was measured 72 hrs post-transfection (n = 3). pcDNA3-miR-128 inhibits luciferase expression from pFLuc-128.2X, indicative of miRNA expression. RLU  =  relative light units.
(EPS)

Figure S5 A. Dose-dependent response to GCV treatment. U373 and A172 cells were transfected in 6-well plates with 1 μg pcDNA3-HSV-TK-128.2X and 1 μg filler DNA (Lipofectamine). 24 hrs post-transfection, cells were plated at 2×10^4^ cells per well in 24-well plates and treated with indicated concentrations of GCV. Crystal violet staining was performed 8 days post-GCV. B and C. U373 cells were co-transfected with 1 μg pcDNA3 (vector), pcDNA3-HSV-TK (HSV-TK), or pcDNA3-HSV-TK-128.2X (HSV-TK-128.2X) and 1 μg either pcDNA3 (vector) or pcDNA3-miR-128 (miR-128). 24 hrs post-transfection, cells were plated in 24-well plates at 1×10^4^ cells per well in media containing 10 μg/ml GCV (B) or as indicated (C). Cells were stained 10-12 days post-transfection. D. Cell viability was determined by trypan blue exclusion. U373 cells were co-transfected with pcDNA3 (vector), pcDNA-HSV-TK, or pcDNA-HSV-TK-128.2X and either pcDNA3 or pcDNA3-miR-128. 24 hrs post-transfection, cells were plated at 2×10^4^ cells per well in 12-well plates and cultured in the presence of 20 μg/ml GCV (n = 2). 10 days post-transfection, viable cells were trypsinized and counted using a hemocytometer.
(EPS)
