# Supplementary material for: Reduced Expression of Brain-Enriched microRNAs in Glioblastomas Permits Targeted Regulation of a Cell Death Gene
Source: PLoS One. 2011 Sep 2;6(9):e24248. doi: 10.1371/journal.pone.0024248 (PMC3166303; doi:10.1371/journal.pone.0024248)
Supplement: Table S1 — Sequenced brain samples. (DOC) [file pone.0024248.s010.doc]

**Table S1. Sequenced Brain Samples.**

| **Sample** | **Brain Origin** | **Diagnosis** | **No. Processed Reads** | **No. Mapped to miRBASE v 16.0** |
| --- | --- | --- | --- | --- |
| N4577 | Temporal, Right | Cortical dysplasia | 2568578 | 2081075 |
| N3034 | Temporal, Right | Cortical dysplasia | 2043911 | 1215551 |
| N3032 | Temporal, Right | Cortical dysplasia | 2649103 | 2194068 |
|  |  |  |  |  |
| G3508 | Parietal, Left | Glioblastoma | 3825267 | 976788 |
| G3183 | Frontal, Left | Glioblastoma | 5280234 | 4429075 |
|  |  |  |  |  |
| G3182 | Temporal, Left | Glioblastoma | 4412128 | 3301057 |
| G3346 | Frontal, Right | Glioblastoma | 4834652 | 2280552 |
|  |  |  |  |  |
| G3522 | Occipital, Right | Glioblastoma | 4220959 | 2577966 |
| G4562 | Occipital, Right | Glioblastoma | 4504220 | 2739927 |
