# Supplementary material for: Reduced Expression of Brain-Enriched microRNAs in Glioblastomas Permits Targeted Regulation of a Cell Death Gene
Source: PLoS One. 2011 Sep 2;6(9):e24248. doi: 10.1371/journal.pone.0024248 (PMC3166303; doi:10.1371/journal.pone.0024248)
Supplement: Table S9 — Oligonucleotides used in this study for deep sequencing, primer extension, and cloning. (DOC) [file pone.0024248.s018.doc]

**Table S9. Oligonucleotides used in this study for deep sequencing, primer extension, and cloning.**

| **Oligo Name** | **Sequence** | **Purpose** |
| --- | --- | --- |
| Sequencing 5' adaptor | GTTCArGrArGrUrUrCrUrArCrArGrUrCrCrGrArCrGrArUrCrNrNrNrNrN* | Deep Sequencing |
| Sequencing 3' adaptor | 5rAppTCGTATGCCGTCTTCTGCTTGT3ddC | Deep Sequencing |
| Sequencing RT primer | CAAGCAGAAGACGGCATACGA | Deep Sequencing |
| Sequencing PCR primer | AATGATACGGCGACCACCGACAGGTTCAGAGTTCTACAGTCCGA | Deep Sequencing |
| PE-miR-124 | TGGCATTCACCGCGTGC | Primer Extension |
| PE-miR-128 | AAAGAGACCGGTTCAC | Primer Extension |
| PE-miR-132 | CGACCATGGCTGTAGA | Primer Extension |
| PE-miR-21 | CAGTCAACATCAGTCTGAT | Primer Extension |
| miR-128-1 FWD | GGCTCGAGTTGCAATAATTGGCCTTG | Cloning pcDNA3-miR-128 |
| miR-128-1 REV | CGTCTAGAAGAAGCCAGGAAGCAGC | Cloning pcDNA3-miR-128 |
| HSV-TK HindIII FWD | GCAAGCTTATGGCTTCGTACCCCTGCCA | Cloning pcDNA3-HSV-TK |
| HSV-TK EcoRI REV | GCGAATTCTCAGTTAGCCTCCCCCACTCC | Cloning pcDNA3-HSV-TK |
| HSV-TK NheI FWD | CAGCTAGCATGGCTTCGTACCCCTGCCA | Cloning pLC-HSV-TK |
| HSV-TK XbaI REV | GCTCTAGACTCGAGTCAGTTAGCCTCCCCCACTCC | Cloning pLC-HSV-TK |
| miR-128 binding sites (TOP) | TCGAGAAAGAGACCGGTTCACTGTGAGAATTCAAAGAGACCGGTTCACTGTGAT | miR-128 binding sites |
| miR-128 binding sites (LOW) | CTAGATCACAGTGAACCGGTCTCTTTGGATCCTCACAGTGAACCGGTCTCTTTG | miR-128 binding sites |

*N = molecular barcode (TTGGC, GCCTA, or ATGC)
